# Fatty infiltration of the pancreas: a systematic concept analysis

**DOI:** 10.3389/fmed.2023.1227188

**Published:** 2023-09-22

**Authors:** Mueataz A. Mahyoub, Mohamed Elhoumed, Abdulfatah Hassan Maqul, Maged Almezgagi, Mustafa Abbas, Yang Jiao, Jinhai Wang, Mohammed Alnaggar, Ping Zhao, Shuixiang He

**Affiliations:** ^1^Department of Gastroenterology, The First Affiliated Hospital of Xi'an Jiaotong University, Xi'an, China; ^2^Clinical Medical Research Center for Digestive Diseases (Oncology) of Shaanxi Province, Xi'an, China; ^3^Department of Gastroenterology, The Second Affiliated Hospital of Xi'an Jiaotong University, Xi'an, China; ^4^Department of Gastroenterology, Faculty of Medicine, Thamar University, Dhamar, Yemen; ^5^Department of Epidemiology and Biostatistics, School of Public Health, Xi'an Jiaotong University Health Science Center, Xi'an, Shaanxi, China; ^6^National Institute of Public Health Research (INRSP), Nouakchott, Mauritania; ^7^Department of Medical Imaging, The First Affiliated Hospital of Xi'an Jiaotong University, Xi'an, China; ^8^Department of Medical Imaging, Sahan Diagnostic Center, Mogadishu, Somalia; ^9^The Key Laboratory of High-altitude Medical Application of Qinghai Province, Xining, Qinghai, China; ^10^Department of Immunology, Qinghai University, Xining, Qinghai, China; ^11^Department of Medical Microbiology, Faculty of Sciences, Ibb University, Ibb, Yemen; ^12^Department of Internal Medicine, Faculty of Medicine, Thamar University, Dhamar, Yemen; ^13^Department of Endocrinology, The Second Affiliated Hospital of Xi'an Jiaotong University, Xi'an, China; ^14^Department of Oncology, South Hubei Cancer Hospital, Xianning, Hubei, China; ^15^Department of Internal Medicine, Clinic Medical College, Hubei University of Science and Technology, Xianning, Hubei, China

**Keywords:** fatty infiltration of the pancreas, metabolic syndrome, obesity, type 2 diabetes mellitus, pancreatic duct adenocarcinoma

## Abstract

Fatty infiltration of the pancreas (FIP) has been recognized for nearly a century, yet many aspects of this condition remain unclear. Regular literature reviews on the diagnosis, consequences, and management of FIP are crucial. This review article highlights the various disorders for which FIP has been established as a risk factor, including type 2 diabetes mellitus (T2DM), pancreatitis, pancreatic fistula (PF), metabolic syndrome (MS), polycystic ovary syndrome (PCOS), and pancreatic duct adenocarcinoma (PDAC), as well as the new investigation tools. Given the interdisciplinary nature of FIP research, a broad range of healthcare specialists are involved. This review article covers key aspects of FIP, including nomenclature and definition of pancreatic fat infiltration, history and epidemiology, etiology and pathophysiology, clinical presentation and diagnosis, clinical consequences, and treatment. This review is presented in a detailed narrative format for accessibility to clinicians and medical students.

## 1. Introduction

Pancreatic fat infiltration was first reported in the scientific literature almost a century ago; however, despite the extensive research on FIP, much about this condition remains unknown. It is, therefore, imperative to regularly review the literature on pancreatic fat infiltration, with a particular emphasis on the diagnosis, consequences, and management of the disease.

During the last decade, pancreatic fat infiltration has been established as a risk factor for various disorders, including type 2 diabetes mellitus (T2DM), pancreatitis, pancreatic fistula (PF), metabolic syndrome (MS), and pancreatic duct adenocarcinoma (PDAC) (1).

In addition, the lack of consensus on the terminology used to describe this condition has led to confusion and inconsistent reporting of research findings. To address this issue, this review adopts the term “fatty infiltration of the pancreas” (FIP) to promote consistency in reporting and facilitate communication between healthcare professionals. Given the interdisciplinary nature of FIP research, this review encompasses a broad range of healthcare specialists, including gastroenterologists, endocrinologists, radiologists, general practitioners, and others.

This review focuses on key aspects of FIP, such as history and epidemiology, etiology, pathophysiology, clinical presentation and diagnosis, clinical consequences, and treatment, aiming to accelerate research progress and translate findings into routine clinical practice. To ensure accessibility for clinicians and medical students, the information is presented in a detailed narrative format.

## 2. Nomenclature and definition of pancreatic fat infiltration

Pancreatic fat is commonly present in humans, with an average weight of 9.7 ± 6.5 g, constituting about 3% of the pancreas' weight in thin individuals. Normal pancreatic fat makes up ~5.5% of the pancreas' weight, primarily consisting of triglycerides (47%), free fatty acids (10%), and total cholesterol (4%). Oleic and palmitic acids are the main triglyceride components, while palmitic and stearic acids are the major free fatty acids (2).

FIP is defined by the presence of one or more of the following features: (1) inter-lobular fat, which is the presence of cells containing fat between pancreatic lobules; (2) lipid droplets within pancreatic acinar cells or islets; (3) pancreatic acinar-to-adipocyte trans-differentiation; and (4) fat replacement of dead pancreatic acinar cells. Notably, FIP excludes peri-pancreatic fat, also called extra-lobular fat (2, 3).

The lack of clear differentiation between triglyceride accumulation in acinar cells, beta cells, or intra-pancreatic adipose cell infiltration has led to various synonyms, such as pancreatic steatosis, pancreatic lipomatosis, or fatty pancreas, to describe all forms of pancreatic fat accumulation outlined in Table 1.

**Table 1:** Nomenclature and definition of pancreatic fat infiltration (2, 3).

**Nomenclature**	**Definition**
FIP IFPD FP	Broad terminology can be employed to describe all types of pancreatic fat accumulation, with the exception of peri-pancreatic (extra-lobular) fat.
FRP	Injury to pancreatic acinar cells results in their demise, leading to their replacement in the pancreas by adipocytes.
PS	The pathological build-up of lipid droplets within pancreatic cells.
NAFPD	Pancreatic adiposity occurs in correlation with obesity and metabolic syndrome.
NAFSP	Pancreatitis is attributed to pancreatic fat accumulation.
LP^*^	A benign, homogenous mass in the pancreas consisting of mature adipose tissue with a well-defined border but no well-formed capsule.
PL^*^	A benign, homogenous mass in the pancreas consisting of mature adipose tissue with a well-defined border and a fibrous capsule.

FIP, fatty infiltration of the pancreas; IPFD, intra-pancreatic fat deposition; FP, fatty pancreas; FRP, fatty replacement of pancreas; PS, pancreatic steatosis; NAFPD, non-alcoholic fatty pancreas disease; NAFSP, non-alcoholic fatty steato-pancreatitis; LP, lipomatous pseudohypertrophy; PL, pancreatic lipoma.

* It does not include the term “fatty infiltration of the pancreas.”

## 3. History and epidemiology

FIP was first described by Oligvie in 1933, who noted that the presence of pancreatic fat was higher in obese individuals (17%) than in those who were thin (9%) (6). In 1926, a biometric analysis was conducted to measure the average pancreatic weight in mature human individuals. It was found that FIP is more prevalent in overweight individuals than in those of normal weight. In adults, the average weight of the pancreas is positively correlated with normal body weight in both males and females, with a stronger correlation observed in females (18). In 1978, a significant description of FIP was completed when 394 cadavers were studied, and it was observed that the proportion of pancreatic fat tends to increase with age (19).

The epidemiology of FIP in the general population needs to be better established. FIP prevalence varies significantly based on the ethnicity of the population and the diagnostic criteria used (see Table 2). A meta-analysis of 11 studies involving a total of 12,675 individuals reported an FIP prevalence of 33% (95% CI: 24%−41%) (20).

**Table 2:** Prevalence of FIP by different methodologies in different countries and ethnic groups.

**Population (n)**	**Prevalence (%)**	**Country**	**Diagnostic method**	**Study design**	**Reference**
293	61.4	South Korea	TUS	Cross-sectional	Lee et al. (4)
230	27.8	USA	EUS	Prospective evaluation	Sepe et al. (5)
557	12.9	Taiwan, China	TUS	Case–control	Wu et al. (6)
8,097	16	China	TUS	Cross-sectional	Wang et al. (7)
685	16.1	Hong Kong, China	MRI and PMRS	Cross-sectional	Wong et al. (8)
901	35	Indonesia	TUS	Cross-sectional	Lesmana et al. (9)
121	47.9	Italy	TUS	Cross-sectional	Della et al. (10)
1,190	30.7	China	TUS	Cross-sectional	Zhou et al. (11)
232	10	USA	CT	Cross-sectional	Pham et al. (12)
12,675	33	Meta-analysis	Various	Meta-analysis	Singh et al. (13)
2,093	2.7	China	TUS	Cross-sectional	Wang et al. (14)
4,419	11	China	TUS	Cross-sectional	Weng et al. (15)
228	25.9	Iran	EUS	Cross-sectional	Sotoudehmansh et al. (16)
4,704	1.2	China	EUS	Retrospective cohort	Chen et al. (17)

TUS, transabdominal ultrasonography; EUS, endoscopic ultrasonography; PMRS, proton-magnetic resonance spectroscopy.

## 4. Etiology and pathophysiology of FIP

**Figure 1:**
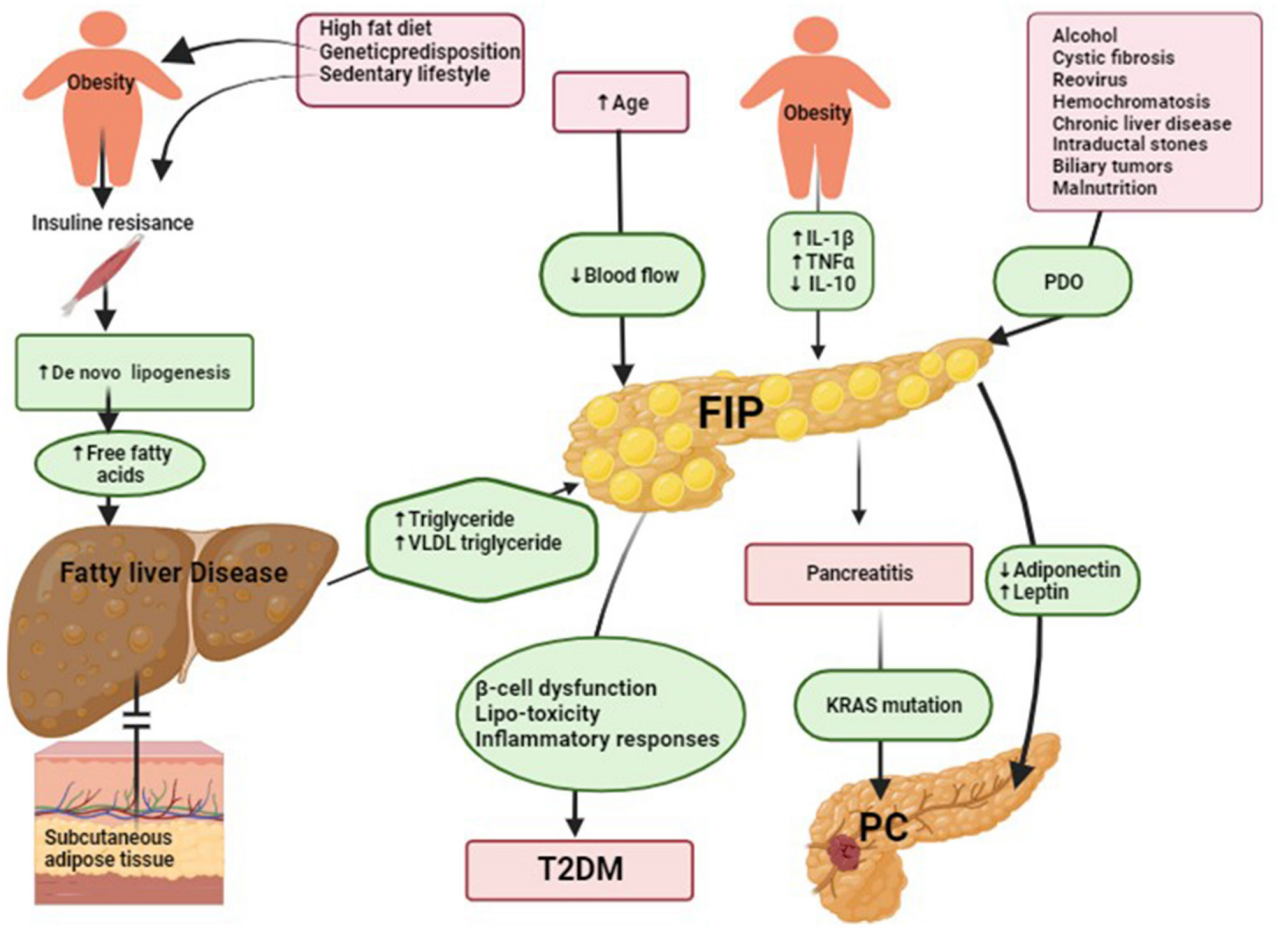
Suggested underlying mechanisms of fatty infiltration of the pancreas (FIP) and its role in type 2 diabetes mellitus (T2DM) and pancreatic cancer (PD) development. (1) Suggested underlying mechanisms of FIP. Insulin resistance reduces cellular responsiveness to insulin, leading to impaired glucose uptake, particularly in muscle and adipose tissue. Various factors contribute to its development, including genetic predisposition, obesity, a sedentary lifestyle, and dietary habits. In individuals with insulin resistance in muscle, glucose uptake by muscle cells is diminished, causing a significant portion of dietary carbohydrates to be redirected toward the liver (21). Excess glucose in the liver undergoes de novo lipogenesis (DNL) to be converted into fatty acids and then triglycerides, contributing to hepatic fat build-up. Additionally, when dietary carbohydrate intake exceeds immediate energy demands, surplus glucose is converted into fatty acids through hepatic DNL, further promoting hepatic lipid deposition. Over time, the progressive build-up of fat in the liver may lead to fatty liver disease. In fatty liver disease, the liver's production of triglycerides is elevated, leading to an increase in plasma very-low-density lipoprotein (VLDL) triglycerides. If subcutaneous adipose tissue cannot adequately store the excess triglycerides, excess ectopic fat accumulates in various tissues, including the pancreas (22). Notably, pancreatic fat build-up occurs through one or more of the following patterns: (1) inter-lobular fat, characterized by the existence of adipose-laden cells positioned amidst pancreatic lobules; (2) lipid droplets residing within pancreatic acinar cells or islets; (3) pancreatic acinar-to-adipocyte trans-differentiation, denoting the intricate process of acinar cell conversion into adipocytes; and (4) adipose replacement of deceased pancreatic acinar cells. Obesity induces chronic low-grade inflammation, increasing pro-inflammatory cytokines such as interleukin-1β (IL-1β) and tumor necrosis factor-alpha (TNF-α). This inflammation leads to higher levels of triglycerides, free fatty acids, cholesterol, and total fat accumulation in the pancreas. Additionally, obesity disrupts the balance of cytokines by reducing the production of the spleen's anti-inflammatory cytokine interleukin-10 (IL-10) (23). These imbalances contribute to FIP, PDO, and pancreatic duct obstruction. (2) Suggested underlying mechanisms of FIP in T2DM. The build-up of ectopic fat has the potential to give rise to a phenomenon termed lipotoxicity, characterized by adverse implications for cellular functionality and metabolic processes. Specifically, within the pancreatic milieu, the manifestation of lipotoxicity assumes a pivotal role in inducing impediments to both insulin secretion and overall cellular performance. Notably, lipotoxicity's ramifications extend to the emergence of insulin resistance upon ectopic fat accrual within the pancreatic tissue, thereby fostering a pro-inflammatory milieu in these domains. The confluence of lipotoxicity, insulin resistance, and localized inflammatory responses collectively perturbs the intricate glucose metabolism homeostasis, thereby initiating disruptions conducive to the genesis of glucose metabolic irregularities. These perturbations, in essence, denote anomalies in regulating blood glucose levels. As temporal progression ensues, these perturbations can evolve into the etiopathological basis of T2DM. Furthermore, the build-up of fat within pancreatic endocrine cells also emerges as a relevant factor potentially contributing to the failure of beta cells and, consequently, assuming a pronounced role in the pathogenesis of T2DM (2). (3) Suggested the underlying mechanisms of FIP in pancreatic cancer (PC) development. FIP plays a pivotal role in promoting PC development. This process involves the secretion of adipokines, including adiponectin and leptin, which drive carcinogenesis through various mechanisms. Increased fat content reduces adiponectin levels and increases leptin levels, contributing to a higher risk of aggressive PC. Adiponectin typically promotes apoptosis and inhibits the availability of growth factors, while leptin activates JAK2 and causes phosphorylation of STAT3, leading to increased transcriptional upregulation of genes involved in angiogenesis, inflammation, anti-apoptosis, repression of interferon-inducible genes, and cell migration and invasion. FIP also leads to the release of pro-inflammatory chemokines and cytokines, fostering a chronically inflamed tumor microenvironment. This environment further supports the progression of PC by influencing cell transition, inflammation, and fibrosis development. Pro-inflammatory signaling, particularly involving cytokines like IL-6, produced by cancer-associated fibroblasts and PC cells, encourages tumor cell invasion and migration. In addition to adipokine secretion, FIP contributes to the dysregulation of cytokines in the tumor microenvironment. The presence of anti-inflammatory cytokines like IL-10 might be an immune response to combat cancer, while pro-inflammatory cytokines and chemokines fuel inflammation and cell transition. The nutrient-deprived tumor microenvironment resulting from FIP can lead to dysfunction in regulatory immune cells that would otherwise promote the apoptosis of developing PC cells. This impaired apoptosis may create a cycle where surviving intra-pancreatic CD8^+^ T cells downregulate key enzymes, ultimately aggravating the accumulation of harmful long-chain fatty acids, which can lead to lipotoxicity. The build-up of lipophilic toxins in pancreatic adipose tissue further exacerbates this process, potentially driving PC development (24).

There are various etiologies associated with FIP (Figure 1), which can be broadly classified into 11 categories:

### 4.1. Demographic determinants

The prevalence of FIP exhibits an age-dependent pattern, with higher rates observed among the middle-aged and elderly populations, as reported in previous studies (19, 25, 26). Notably, the onset of the fifth decade appears to be a critical time point when an increase in pancreatic fat fraction is commonly observed (27). Furthermore, it has been demonstrated that middle-aged men are at a higher risk of developing FIP than their younger counterparts (8). FIP also increases in middle-aged and elderly women compared to young women, and the risk ratio was 6.60 (95% CI: 0.92–47.49) and 19.11 (95% CI: 2.57–142.28), respectively. Post-climacteric women have a higher risk of FIP than pre-climacteric women (28). The cause of age-related pancreatic fat build-up is unclear, but atherosclerosis and reduced blood flow may contribute (29). FIP is more prevalent in individuals of East/Southeast/South Asian heritage, less common in Black African individuals, and intermediate in European or Latin American individuals (30).

### 4.2. Obesity and metabolic factors

Adipose tissue is an endocrine organ that communicates with other organs in the body and contains adipocytes and other tissue cells. When body weight increases, adipose tissue storage capacity is exceeded, leading to a relocation of fat in non-adipose organs, such as the liver, skeletal muscle, and pancreas (21). In obesity, a hormonal microenvironment disorder causes an increase in macrophage infiltration into fat tissue. This leads to a chronic low-inflammatory state characterized by pro-inflammatory cytokine release and fat accumulation in distal organ tissues (23, 31). In animal studies, obese mice with leptin insufficiency had a higher pancreas weight. They exhibited greater intra-lobular and total pancreatic fat content and elevated levels of pro-inflammatory mediators such as tumor necrosis factor-alpha (TNF-α) and interleukin-1β (IL-1β).

Additionally, pancreatic tissue triglyceride composition was significantly higher in the progeny of obese dams than in the progeny of thin dams, indicating that exposure to an obesogenic environment shortly after birth can lead to FIP. The study revealed a previously unknown fatty pancreatic phenotype associated with obesity, which suggests that maternal obesity can program a pancreatic phenotype similar to non-alcoholic fatty liver disease (NAFLD) (32). Obesity and insulin resistance play essential roles in pancreatic adipocyte infiltration, which can lead to FIP (22). Although old studies have reported T2DM as the cause of FIP (5, 33), recent studies have found that FIP is associated with T2DM development (34, 35). FIP can often be cured by reducing weight (36).

### 4.3. Diet

Diet may play a pivotal role in initiating FIP in experimental animal models. In a rat model, prolonged exposure to a high-fat diet resulted in adipose tissue build-up within pancreatic acinar cells, triggering a cascade culminating in tissue fibrosis and injury (37). Similar observations were made in mice fed a high-fat diet, which displayed FIP characteristics and MS markers, including insulin resistance (38). The impact of high-fat diets on pancreatic physiology appears to involve intricate interactions between saturated and unsaturated fatty acids along with overall fat content. This suggests that dietary fats' specific composition and quantity influence pancreatic impairment (39). Oleic acid, an unsaturated fatty acid, emerged as a determinant influencing individual variations in pancreatic triglyceride accumulation, underscoring the significance of dietary fat composition (40).

In humans, a study involving 11 individuals with T2DM revealed that a low-calorie diet significantly reduced pancreatic fat content (FIP) by creating a negative energy balance, implying its relevance to T2DM-associated pancreatic fat (41). A trial comparing Mediterranean (rich in unsaturated fats) and low-fat diets among individuals with abdominal obesity or dyslipidemia, including T2DM cases, reported significantly lower FIP prevalence with the Mediterranean diet (42). These findings underscore the potential of diet to influence pancreatic fat content and metabolic health, particularly in individuals with T2DM.

A prospective study involved three distinct cohorts, totaling 120,877 US women and men, all of whom were initially free from chronic illnesses and not obese. The follow-up periods extended from 1986 to 2006, 1991 to 2003, and 1986 to 2006. In each successive 4-year interval, participants exhibited an average weight gain of 3.35 lbs, ranging from the 5th to the 95th percentile (−4.1 to 12.4 lbs). When examining augmented daily servings of individual dietary constituents, the alteration in weight over 4 years was most notably linked to the consumption of potato chips (1.69 lb), potatoes (1.28 lb), sugar-sweetened beverages (1.00 lb), unprocessed red meats (0.95 lb), and processed meats (0.93 lb). Considering obesity's established association with a significant risk for FIP, it is plausible that meat consumption may contribute to the development of FIP (43).

### 4.4. Non-alcoholic fatty liver disease

NAFLD is a condition with excessive fat build-up in hepatocytes and is prevalent in approximately 30% of the general population. FIP has been increasingly recognized as a condition that can occur with NAFLD. A study that included post-mortem material from 80 patients found that total pancreatic fat is a major determinant of the presence of NAFLD. Specifically, intra-lobular pancreatic adipose build-up was associated with non-alcoholic steatohepatitis (NASH), as reported in a prior investigation (22). Furthermore, a study that employed transabdominal ultrasonography (TUS) to assess the relationship between NASH and FIP found that over 50% of individuals with NASH exhibited concurrent FIP. FIP was observed at various stages in 51.2% of NASH patients and 14% of normal individuals (44). In another prospective study, it was observed that nearly 80% of NASH patients also developed FIP. Hepatic steatosis, in particular, was significantly associated with FIP (45).

Moreover, a retrospective study used proton density fat fraction (PDFF) and MRI to evaluate the extent of pancreatic fat in individuals with NAFLD confirmed by biopsy. The average MRI-PDFFs for the liver and pancreas were 18.7% and 5.7%, respectively. T2DM patients had a significantly higher association (12.2% vs. 4.8%) than non-T2DM patients (46).

A systematic review and meta-analysis found that FIP was associated with a more than 2.5-fold higher co-prevalence of NAFLD and a substantial positive correlation between pancreatic and liver fat content in an uncorrected analysis of healthy individuals (13).

### 4.5. Hemochromatosis

Hemochromatosis is a genetic disorder that causes systemic iron overload due to reduced hepcidin or hepcidin-ferroprotein binding levels, leading to iron accumulation in parenchymal cells in the liver, pancreas, and heart (47). In the pancreas, this can cause function dysregulation, fibrosis, and tissue replacement with fat (48). Studies have shown a correlation between hemochromatosis and fat accumulation in the pancreas, which may contribute to the development of FIP (49–51).

### 4.6. Virus

FIP has been associated with hepatitis B, reovirus, and HIV. An autopsy report revealed that a 52-year-old Japanese woman with cirrhosis caused by chronic hepatitis B also had FIP, indicating a possible correlation between chronic hepatitis B or extensive hepatic lesions and FIP (52). An animal study found that after the third week of reovirus infection, there was a fatty replacement of some necrotic tissue in the pancreas, suggesting a potential relationship between reovirus and FIP (34). HIV has also been linked to FIP, which can directly harm the pancreas (34, 52).

### 4.7. Alcohol consumption

Studies suggest that long-term alcohol intake can contribute to the development of FIP. An animal study found that alcohol consumption in the long term promotes pancreatic cholesteryl ester build-up and induces FIP (53). Furthermore, a human histopathological examination has observed fat storage in pancreatic acinar cells in patients who consume more than 30 g/day of ethanol (54). Another study reported that alcohol consumption of more than 14 g/week can lead to FIP (34, 48).

### 4.8. Drug use

Abnormal lipid metabolism induced by Cushing syndrome or steroid therapy has been identified as a contributing factor to the development of FIP. A case report study demonstrated a significant association between the administration of cortisone or its analogs and the occurrence of FIP in affected patients (48). FIP is frequently observed in individuals with HIV-1 undergoing antiretroviral therapy (55).

During the HAART era, FIP has become a prevalent clinical consequence. Reports have indicated that changes in body composition occur with the introduction of HIV protease inhibitors (HAARTs). These changes involve a seemingly increased redistribution of fat, including a relative increase in abdominal fat with peripheral lipoatrophy. These changes caused peripheral lipoatrophy and increased circulating lipids, leading to excess fatty acids deposited in pancreatic cells (56).

According to an animal study, Rosiglitazone leads to FIP by increasing pancreatic bulk, fat entrapment, acinar degeneration, and the invasion of inflammatory cells (57). A clinical case report study of an individual with pancreatic head malignancy found total fat substitution of the pancreas following a chemoradiotherapy regimen that included Gemcitabine and radiotherapy after excluding the fatty replacement in the pancreatic bed before the therapy; hence, Gemcitabine may cause FIP (58).

### 4.9. Pancreatic duct obstruction

PDO has been investigated concerning FIP. Post-mortem and case report studies have reported an association between PDO due to intraductal calculus or pancreatic cancer and the development of FIP (33, 59, 60). However, an animal study showed that simple closure of the pancreatic duct only resulted in fibrosis and not FIP due to insufficient inflammatory cell infiltration. These findings were reversed with pancreatic juice drainage, indicating that more arterial occlusion was necessary to induce FIP (61). Therefore, chronic ischemia caused pancreatic damage, limited cell necrosis, and the formation of a reaction zone, which led to chronic pancreatitis and, ultimately, FIP.

### 4.10. Genetic predisposition

Several genetic abnormalities have been identified as causing FIP, among other conditions. FIP is a late-stage pancreatic disease observed in individuals with cystic fibrosis, and it is primarily caused by a deficiency or malfunction of the cystic fibrosis transmembrane conductance regulator (CFTR) protein in the pancreatic duct epithelium (PDE) (62). The CFTR protein functions as a chloride and bicarbonate channel on the apical surface of epithelial cells, including those in the pancreas. CFTR malfunction leads to reduced bicarbonate secretion from the PDE, causing an acidic small intestine environment, thickened mucus production, and PDO, ultimately resulting in FIP (63).

In Western countries, Schwachman-Diamond syndrome (SDS) ranks second in prevalence among exocrine pancreatic genetic disorders, following cystic fibrosis. The disorder is commonly associated with exocrine pancreatic dysfunction, which can lead to the development of FIP (64, 65).

Johanson-Blizzard syndrome is an uncommon genetic disorder associated with exocrine pancreatic insufficiency (EPI) (66). This disorder is distinguished by congenital EPI, which stems from histological variances in the pancreatic buds during embryonic development and can lead to uneven fatty infiltration (67, 68). Radiological examination of the pancreas in non-diabetic children with carboxyl-ester lipase (CEL) gene mutations revealed structural changes indicating fat build-up. It confirmed previous research on EPI in CEL-mediated disease in all mutant carriers over 5 years old (69).

A recent study on twins found that the impact of hereditary factors on FIP detected by CT was limited. Upon controlling for various factors, the statistical significance of the association with FIP was exclusively detected in monozygotic twins but not in dizygotic twins (70).

### 4.11. Severe malnutrition

The severe malnutrition condition of kwashiorkor may play a role in the development of FIP, according to a case report study of an 11-year-old boy who presented with clinical and pathological features of kwashiorkor for over 2 years and was diagnosed with FIP (34). Therefore, kwashiorkor may contribute to the development of FIP.

## 5. Diagnosis of FIP

Many patients with FIP may not display any noticeable symptoms, and diagnosis often relies on conducting investigative tests, which are summarized in Table 3. Two types of investigative tests for FIP are available: invasive and non-invasive.

**Table 3:** Summary of diagnostic tests of FIP (71, 72).

**Diagnostic modalities**	**Signs of FIP**	**Advantage**	**Disadvantage**
Histopathology	Infiltration of adipocyte and fat deposition intracellularly, including both acinar and islet cells.	High accuracy.	Invasive. Risk of complications.
TUS	Hyperechogenic pancreas.	Widely available. Non-invasive.	Low accuracy. Operator depended.
EUS	Hyperechogenic pancreas.	Superior spatial Resolution, accurate imaging modality (80–90%) (73, 74).	Invasive Risk of complications.
CT	Hypodense.	Easily available. No need for iv-contrasts. Accurate with sensitivity and specificity of 79.3 and 42.4%, respectively (75).	Radiation exposure.
MRI	Hyperintense (white).	High accuracy (76).	Technical challenges. High cost. Long scanning duration. It is susceptible to MR chemical shift artifacts.

TUS, transabdominal ultrasonography; EUS, endoscopic ultrasonography.

### 5.1. Invasive test

#### 5.1.1. Histopathology

**Figure 2:**
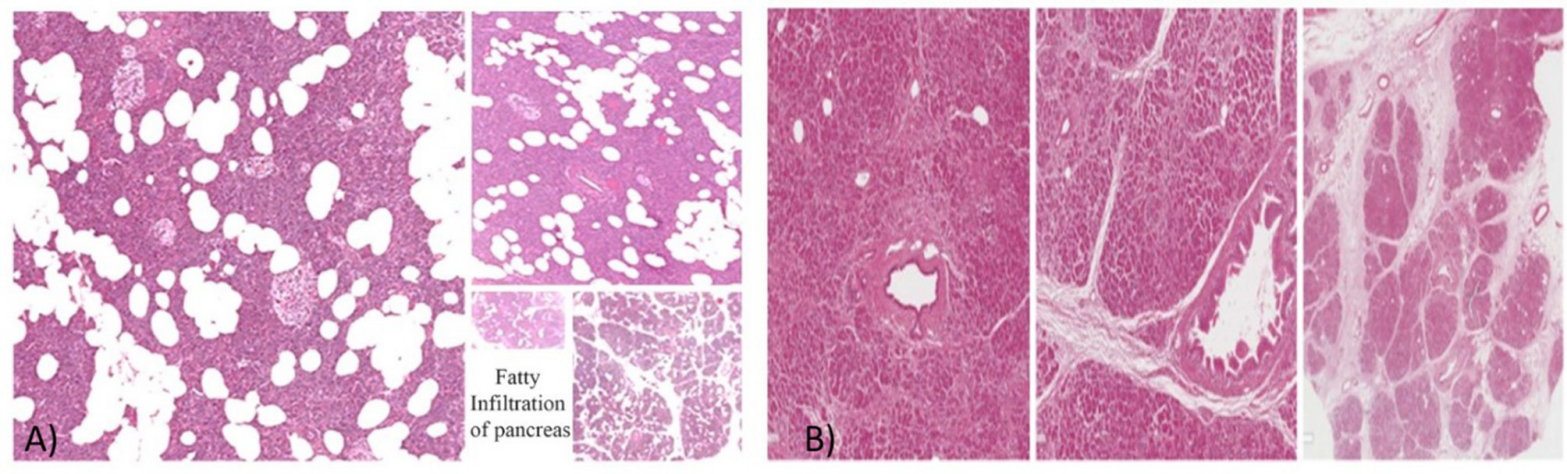
**(A)** The histopathology of FIP. **(B)** The histopathology of a normal pancreas. Courtesy of Prof. Vasquez E. and Dr. Angelico G., Anatomical Pathological Department, University of Catania, Catania, Italy (78).

Histopathology is the gold standard for diagnosing FIP (77). FIP is characterized by adipocyte infiltration and intracellular fat deposition in acinar and islet cells (Figure 2). Collecting a pancreatic biopsy after surgery has limited indications and cannot be widely adopted due to the low frequency of pancreatic surgery among those at risk for FIP. Endoscopic ultrasonography fine needle aspiration (EUS-FNA) biopsy can also be used. Nonetheless, it carries a significant risk of complications and is only considered ethical when differentiating between malignant and benign localized pancreatic tumors (2). The build-up of fat in the pancreas can be either homogeneous or heterogeneous. Heterogeneous FIP is classified into Category 1A (35%), which involves the head, not the uncinate process or the peribiliary area. Category 1B (35%) involves the head, neck, and body but spares the uncinate and peribiliary areas. Category 2A (12%) involves the head and uncinate process but spares the peribiliary area. Finally, Category 2B (18%) involves the entire pancreas replacement but spares the peribiliary area (77).

### 5.2. Non-invasive tests

#### 5.2.1. Biomarker

Currently, there is no reliable biomarker for diagnosing FIP, and histopathology and imaging remain the primary diagnostic methods. However, a meta-analysis study suggests a modest positive correlation between FIP and triglycerides (TG) and a modest negative correlation between FIP and high-density lipoprotein cholesterol (HDL-C) among lipid metabolism markers. Additionally, several glucose metabolic biomarkers have shown a significant link to FIP, including glycated hemoglobin (HbA1c), fast insulin, homeostasis model assessment of insulin resistance (HOMA-IR), and fast glucose. HbA1c may be a useful marker for FIP diagnosis as it is stable, not affected by changes in activity or exercise, and does not require fasting before testing. The pathophysiology of FIP is likely influenced by underlying inflammation. Inflammatory markers, such as high-sensitivity C-reactive protein (CRP) and plasminogen activator inhibitor-1 (PAI1), have minor connections with the contents of pancreatic fat. The liver produces CRP, an acute-phase protein, in response to the pro-inflammatory cytokine IL6. While CRP is a well-known inflammation index, it is not a valid and precise biomarker for FIP. CRP affects plasminogen activator inhibitor 1, a protein that helps coordinate fibrinolysis (20).

#### 5.2.2. Ultrasonography

Two ultrasound modalities, TUS and EUS, can detect FIP, each with advantages and disadvantages. TUS is quick, cheap, and safe (79), but it is insensitive to mild to moderate FIP and may not always visualize the pancreas, especially in obese patients (80). EUS offers detailed imaging of the pancreas and the ability to collect FNA/B, but it is a riskier procedure than TUS. However, both modalities are operator-dependent, and comparing pancreatic echogenicity to hepatic or nephrotic echogenicity is subjective (81).

**Figure 3:**
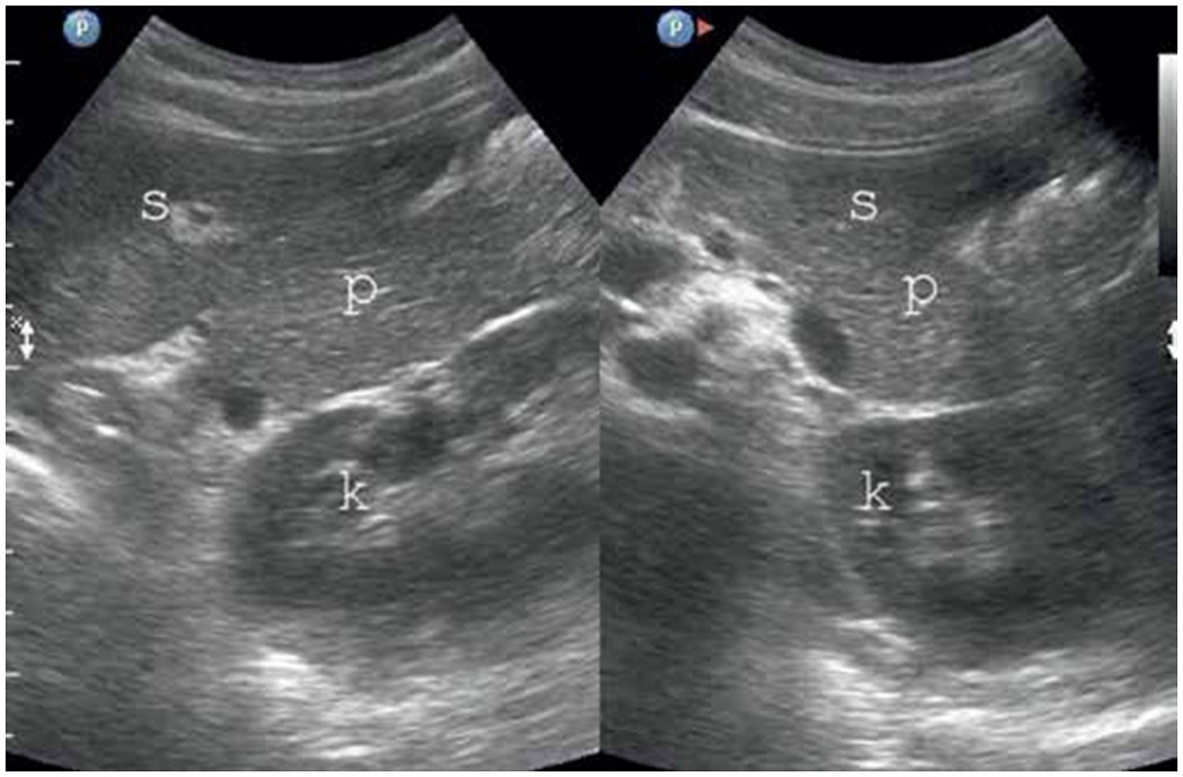
FIP on TUS. The pancreas has increased echogenicity compared to the spleen and kidney (80). P, pancreas; S, spleen; K,kidney.

In TUS, a hyperechogenic pancreas is a diagnostic sign of FIP (Figure 3), but fibrosis or fibro-lipomatosis can also cause a similar sign (82). Various methods have been used to identify FIP in TUS, including comparing the pancreas' echogenicity to other organs such as the liver, renal parenchyma, spleen, and retroperitoneal adipose tissue (45).

Researchers have used the spleen as a comparison organ in EUS to quantify the degree of FIP. They used the ImageJ software to estimate the midpoint value of pancreatic brightness at the adipose surrounding the liver on both the transverse and longitudinal scans. This stratum of adipose tissue is located between an anterior abdominal muscle stratum and an anterior liver surface, and the results are used to calculate the pancreas–liver index (83).

Another study adapted the previous TUS method to evaluate FIP by assessing the entire pancreas using gray-scale imaging on a body cross-section (80). The body of the pancreas was considered to have corrected echogenicity if it had less echogenicity than the retroperitoneal adipose in the region of a superior mesenteric artery (SMA), while the splenic vein (SV) and PD were completely demarcated. The first degree of FIP occurs when the pancreas has echogenicity similar to that of fatty tissue in the region of the SMA, with a uniform, raised echogenicity and a flat abdominal contour, while the SV, SMA, and PD are clearly visible. The second degree of FIP is linked to elevated echogenicity, a darker background in the dorsal part of the pancreas, indistinct borders of the SV and PD, and an almost imperceptible region of the SMA. In some cases, the abdominal contour of the pancreas appears wavy and may exceed the usual limit of visibility. The third degree of FIP is characterized by worsened ultrasonic wave propagation of the pancreas, allowing only its abdominal portion to be visible, often with a clear external waviness, while the SV, SMA, or PD are not visible. The second and third degrees of FIP may entail a reciprocal invasion of the hyperechogenic fatty lobes of the pancreas and the hypoechogenic fatty tissue encircling it, also referred to as interference (84).

#### 5.2.3. Computed tomography

CT is commonly used for imaging abdominal organs and is an excellent option for visualizing the pancreas due to its widespread availability, fast acquisition time, and routine use by clinicians (85).

Although no agreed-upon diagnostic criteria exist for FIP on CT, researchers have used various techniques, such as measuring pancreatic attenuation through non-enhanced CT images with three regions of interest (ROIs) on the head, body, and tail of the pancreas.

**Figure 4:**
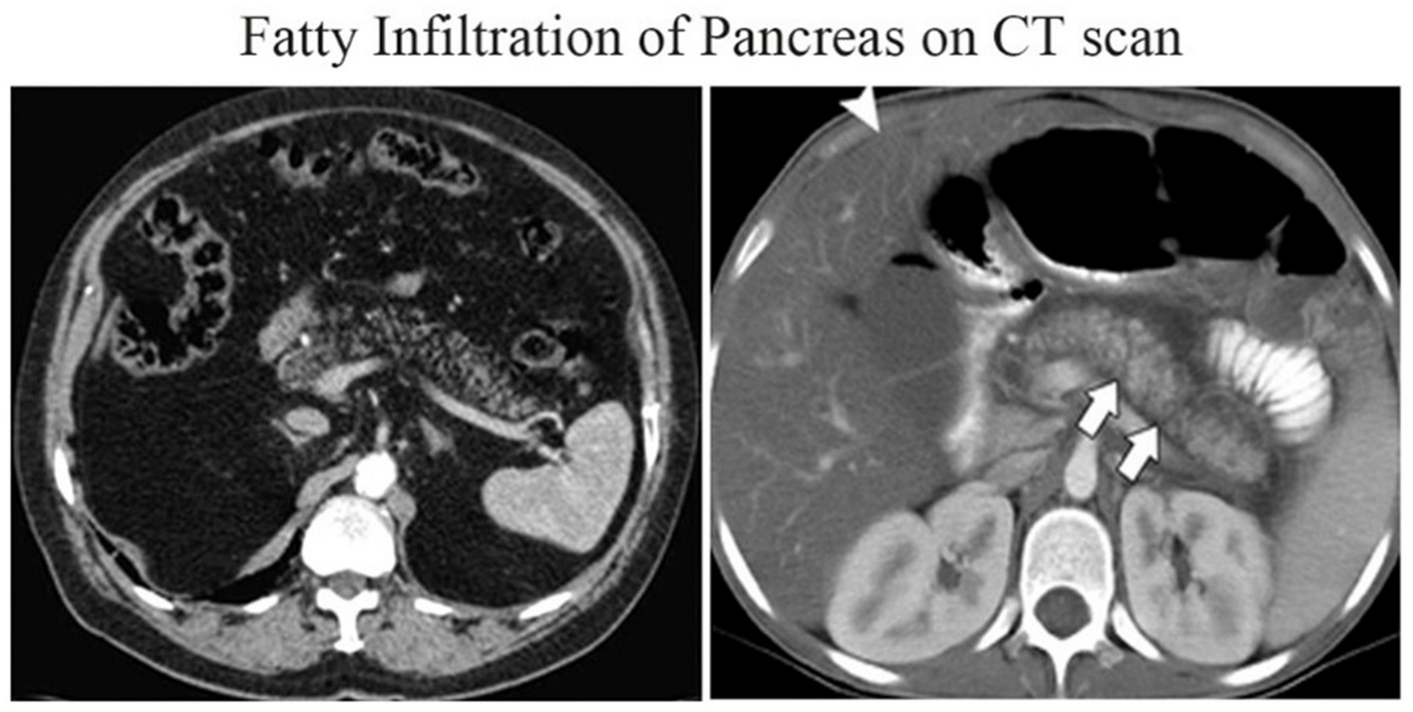
FIP on CT (98).

The theory is that fat accumulation in visceral organs decreases Hounsfield units (HU), making fat visible on CT if there is more pancreatic inter-lobular fat (Figure 4). The attenuation within the ROI can be standardized by adipose attenuation (less than −30 HU) using histogram analysis to obtain a percentile of adipose attenuation values (86).

Studies have shown a good correlation (*r* = 0.67) between CT and histologic measurements of FIP. The variance in CT attenuation between the pancreas and the spleen has also been used to assess FIP. Other volumetric histography research has revealed an average pancreatic fat content of 27.9 cm^3^ and an average fat-to-parenchyma ratio of 0.69. This technique evaluates individual pixel values to quantify FIP (87).

#### 5.2.4. Magnetic resonance spectroscopy (MRS)

**Figure 5:**
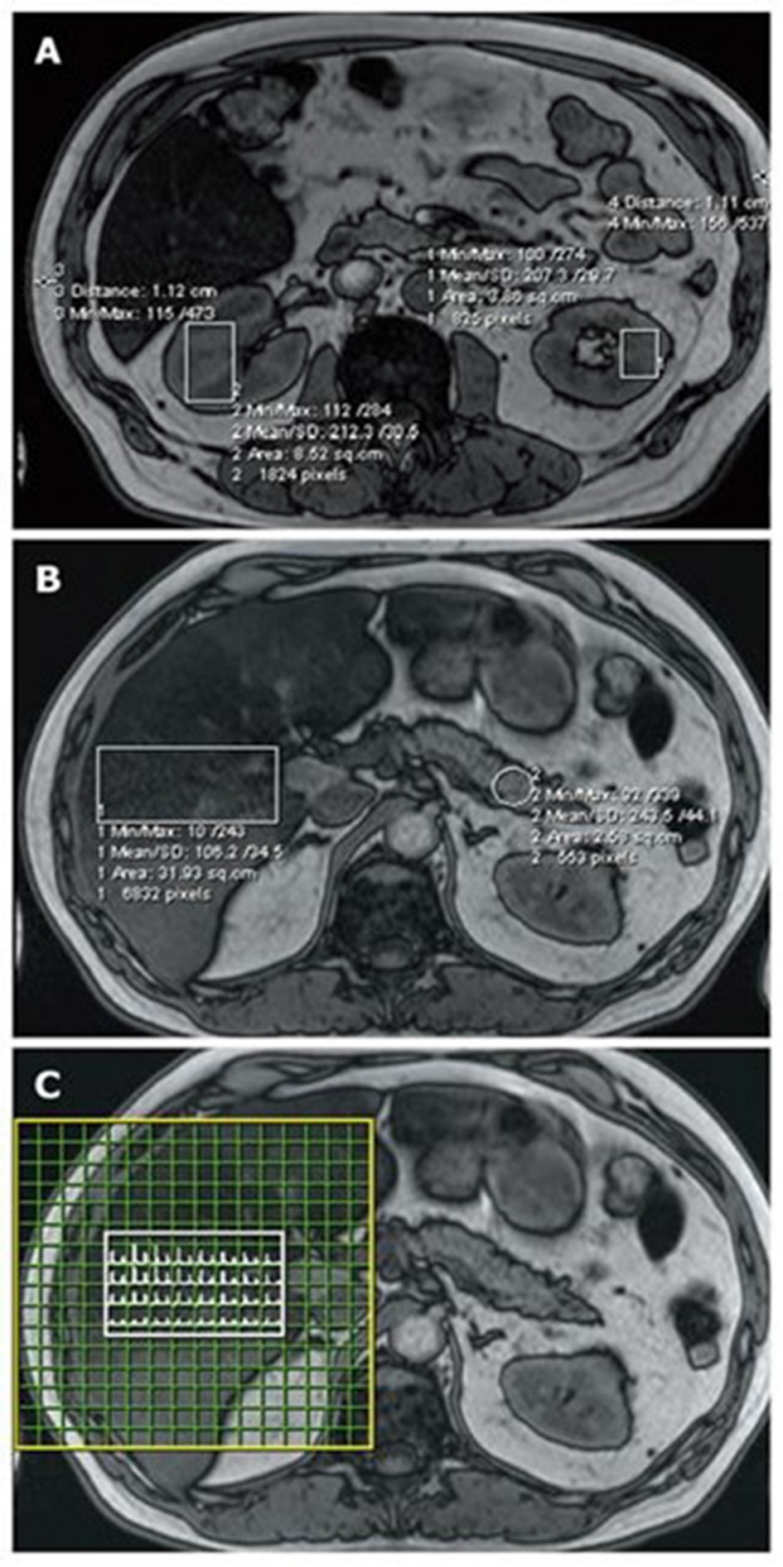
FIP assessment using MRI and MRS. Regions of interest are highlighted on magnetic resonance imaging (MRI) (OP series with a 70° pulse angle and TE = 2.4 ms) and the corresponding magnetic resonance spectroscopy (MRS). **(A)** Kidney and subcutaneous tissue measurements; **(B)** liver and pancreas measurements; and **(C)** the liver MRS spectral map, which shows a range of water and fat peaks (89).

Magnetic resonance spectroscopy (MRS) is the preferred non-invasive method for pancreatic fat assessment (Figure 5). However, using MRS requires manually arranging a voxel (typically 2.0 × 1.0 × 1.0 cm, for example, 2.0 cm^3^) to encompass more pancreatic tissue while avoiding the major PD and blood vessels (7).

MRS presents several technical challenges. Prolonged acquisition times result from the essential requirement for precise local shimming to attain the best possible magnetic field uniformity. Furthermore, when aiming to obtain spectra from small voxels, there is a need for substantial signal averaging during the acquisition process. This increases vulnerability to cardiac motion artifacts from nearby pulsatile vessels and respiratory motion artifacts. Visceral or peritoneal fat outside the pancreas typically contaminates the voxels, lowering shim quality and causing large changes in recorded spectra (88).

However, longer acquisition times can lead to patient discomfort, resulting in patient movements. MRS is operator-dependent and susceptible to noise-induced errors due to the low-fat content in the intra-lobular pancreas. The reproducibility and replicability of pancreatic MRS assessments have been flawed and inferior to those observed in the liver (89).

#### 5.2.5. Chemical shift MRI

MR scanning techniques offer the advantage of setting quantitative data throughout an entire scanned region. However, quicker acquisition times can limit pancreatic fat measurement. Chemical shift imaging (CSI) is a technique that helps overcome this limitation by acquiring an imaging signal at two or more echo times, which allows for contrasting the chemical shift between the primary fat and water peaks (90).

Fat content estimation can be achieved through the subtraction of the “in-phase” image, which depicts the constructive interaction between fat and water, from the “out-of-phase” image, which represents their destructive interaction (27, 91, 92). This technique offers more sophisticated assumptions about fat distribution within the pancreas, surrounding viscera, and other tissues, which may be investigated with faster acquisition times.

#### 5.2.6. Proton density fat fraction mapping

**Figure 6:**
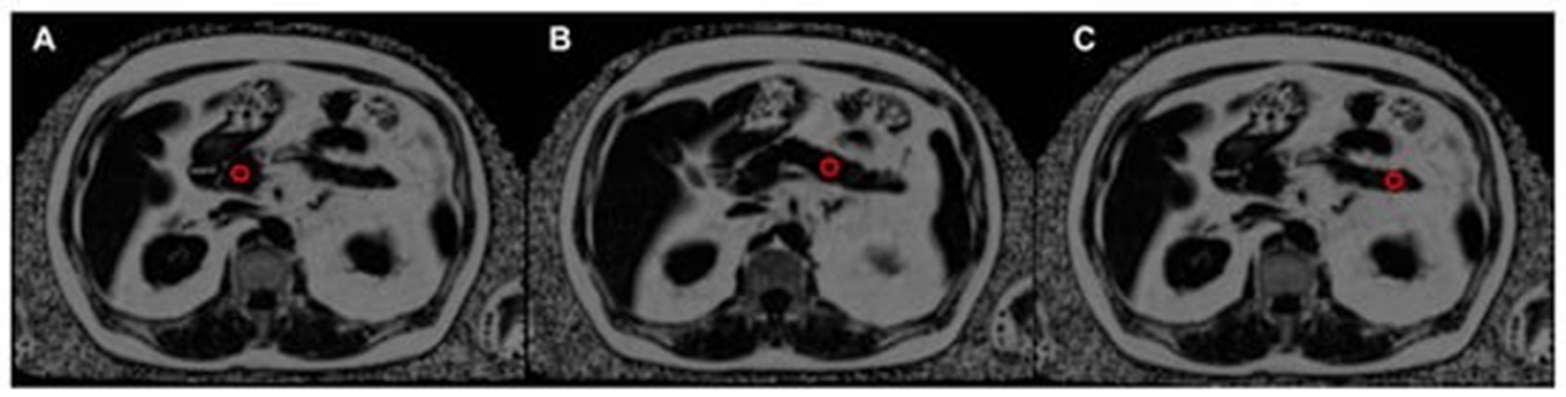
The assessment of pancreatic fat content using a proton density fat fraction (PDFF) map, with the position of three regions of interest (ROIs) indicated by red circles. The ROIs are located in the pancreatic head **(A)**, pancreatic body **(B)**, and pancreatic tail **(C)** (93).

PDFF MRI is currently the most advanced method for estimating fat content via MRI (Figure 6), but it has limitations. PDFF sequences are designed to evaluate hepatic fat, and further advancements are needed to accurately quantify pancreatic fat content. The complexity of the pancreatic fat spectrum has been examined, and current *in vivo* spectra may be susceptible to contamination from extra-lobular fat. Surgical samples for *ex vivo* pancreatic MRS could be a feasible alternative, although they are likely to contain a significant amount of extra-lobular fat, particularly in cases of fatty infiltration (93).

#### 5.2.7. The new FIP diagnostic tool

A research study by Khoury et al. developed a simplified scoring system to identify the presence of FIP. The scoring system is based on the presence of obesity, hyperlipidemia, and fatty liver, each of which is assigned a score of 1, resulting in a maximum score of 3. The scoring system has demonstrated high accuracy and specificity, with a receiver operating characteristic (ROC) value of 0.77 and a specificity range of 82.1%−98.4%. Patients with a score of 1 have a low probability of having FIP, whereas those with a score of 2 or more have a higher likelihood (94).

## 6. Classification of FIP

**Figure 7:**
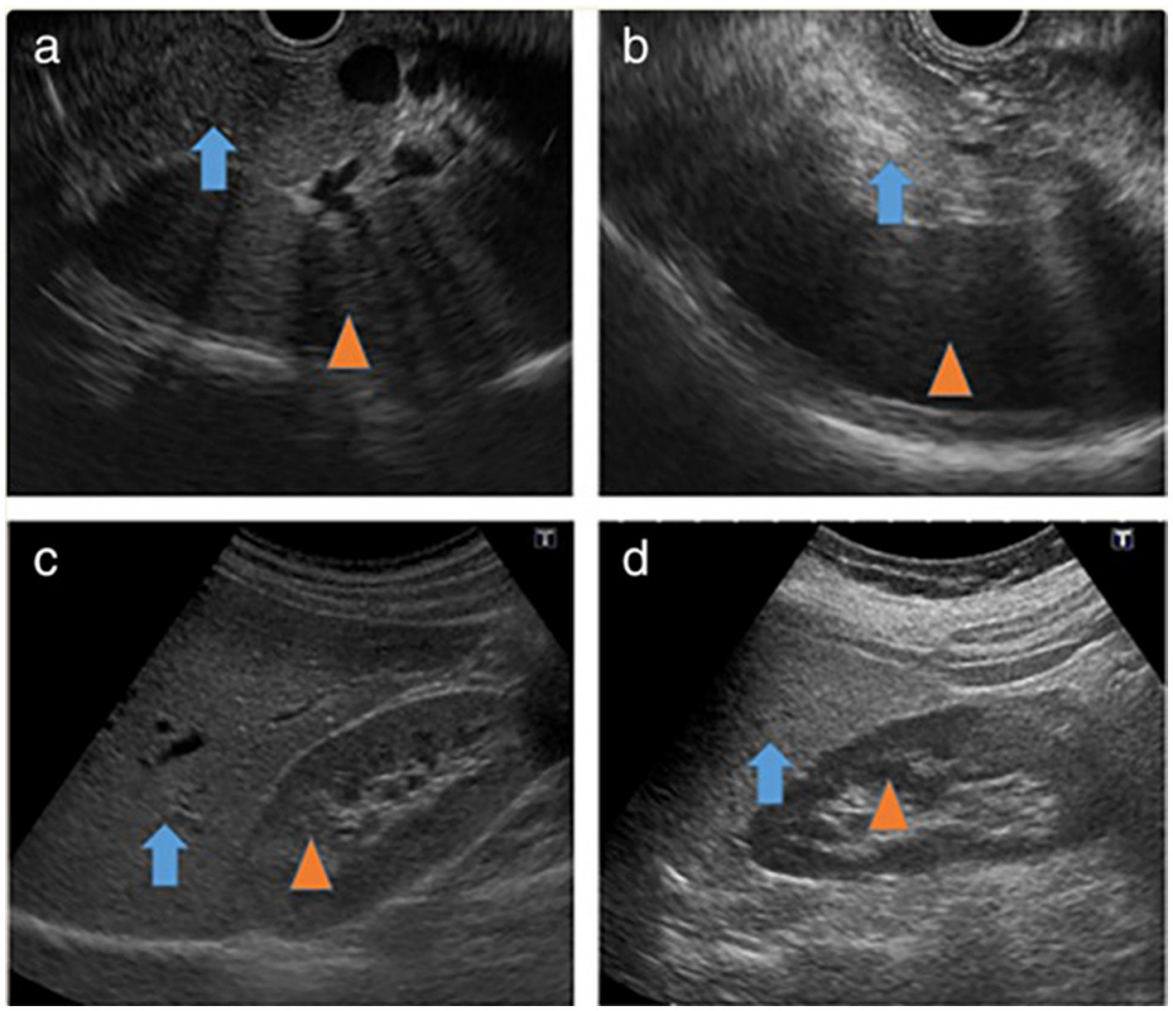
Fatty infiltration of the pancreas on EUS (97). **(a)** Iso-dense image of the normal pancreas (arrow) with the spleen (arrowhead). **(b)** High-density image of fatty infiltration of the pancreas (arrow) with the spleen (arrowhead). **(c)** Iso-dense image of the normal liver (arrow) with the right kidney (arrowhead). **(d)** High-density image of fatty liver (arrow) with the right kidney (arrowhead).

By EUS, the pancreatic adipose tissue classification system was initially established based solely on pancreatic echogenicity (95, 96). Nonetheless, the current assessment version now encompasses an evaluation of the pancreatic parenchyma and the clarity of the PD border (Figure 7).

Grade I pancreatic fat is hypoechoic or isoechoic compared to the spleen, with a well-defined major PD and “salt and pepper” specks visible in the pancreatic parenchyma.

Grade II pancreatic fat is described as having more than 80% of the parenchyma hyperechoic compared to the spleen, with a well-identified major PD and salt and pepper specks visible in the pancreatic parenchyma.

Grade III pancreatic fat has more than 80% more hyperechoic parenchyma than the spleen, with relatively unclear major PD borders and salt and pepper pancreatic parenchyma (5).

Grade IV pancreatic fat is where the pancreas cannot be distinguished from nearby fat, the major PD boundaries are intensely unclear, and salt and pepper specks in the parenchyma of the pancreas are intensely fuzzy. Grades I and II indicate a normal pancreas, while grades III and IV indicate FIP (5).

## 7. Clinical consequences of FIP

**Figure 8:**
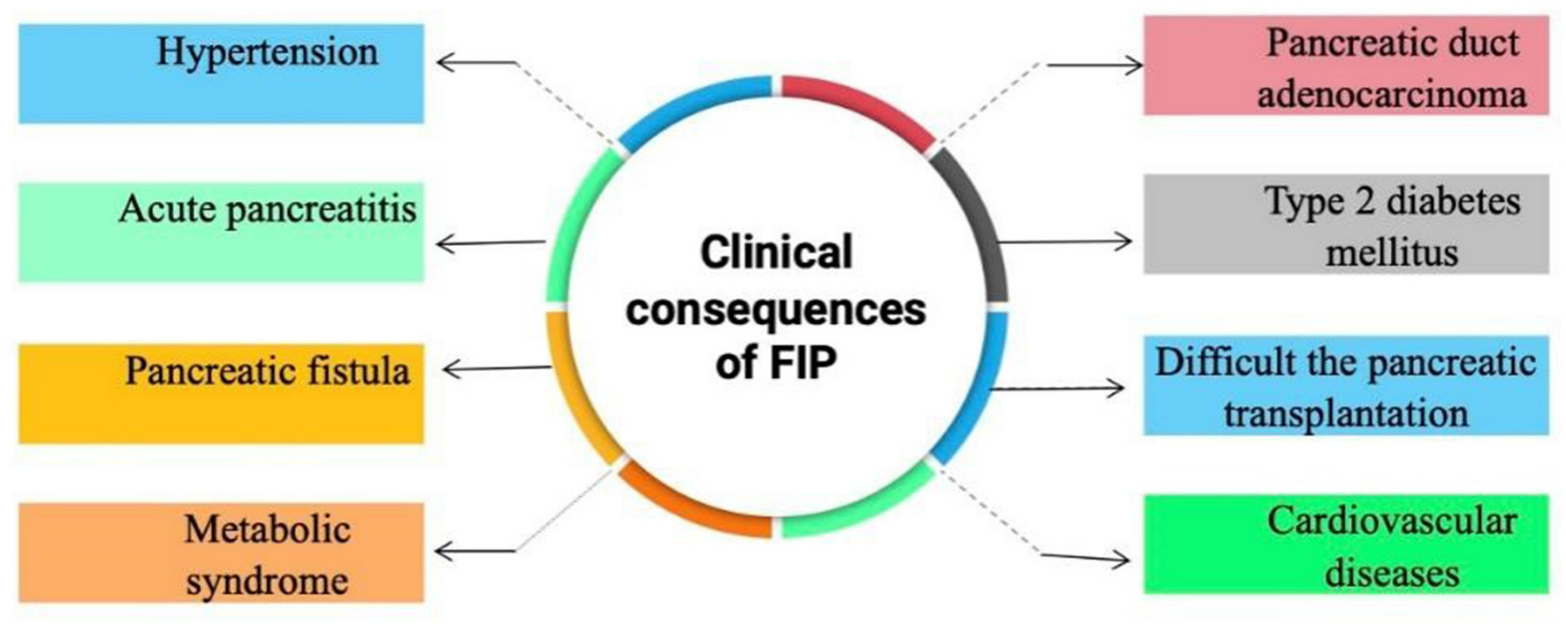
Clinical consequences of FIP.

The clinical consequences of FIP are summarized in Figure 8.

### 7.1. FIP and acute pancreatitis

It has been established that FIP directly impacts the pancreatic parenchyma and is linked with parenchymal damage in acute pancreatitis, specifically in the adipose tissue enveloping the acinar cells of the pancreas (99, 100). A human study revealed that deceased adipocytes were surrounded by an area of necrotic parenchyma, with the most severe damage observed near the dead adipose tissue. This peri-fat acinar necrosis, characterized by the presence of macrophages (CD68-positive), is an antemortem event and was found to be more common in patients with acute pancreatitis, especially those with severe forms of the disease, compared to controls. It was also the most frequently observed type of necrosis in post-mortem samples. Another type of necrosis observed was isolated acinar necrosis, which occurs in pancreatic parenchyma away from the adipose tissue. However, this type of necrosis was significantly less common than peri-fat acinar necrosis in severe acute pancreatitis. The presence of FIP contributes to increased morbidity and mortality in patients with severe acute pancreatitis (101).

### 7.2. FIP and MS

MS is a growing clinical and societal concern worldwide due to changing lifestyles characterized by high-calorie, high-fat diets and low physical activity levels. A recent study revealed that the group with FIP had a significantly higher prevalence of MS than the control group, along with a greater number of MS markers (*P* < 0.05). Furthermore, FIP showed a strong association with MS, as evidenced by four studies involving 611 individuals with FIP, of whom 265 had MS, compared to 2,051 non-FIP individuals, of whom 361 had MS. The presence of FIP was significantly correlated with a higher incidence of MS, with a relative risk of 2.37 (95% CI: 2.07–2.71; *P* < 0.001), indicating a 137% increased likelihood of developing metabolic syndrome in individuals with FIP (20).

In addition, a prospective study demonstrated that patients with FIP were over three times more likely to receive an MS diagnosis than those without FIP (OR 3.13, *P* = 0.004). This study also explored the relationship between FIP and increasing MS risk factors and whether a higher BMI could largely explain the correlation between FIP and MS. The findings revealed that each additional MS risk factor, such as BMI >30 kg/m^2^, T2DM, HTN, or hyperlipidemia, increased the risk of FIP by 37% (5).

### 7.3. FIP and cardiovascular diseases

FIP has been implicated in the development of cardiovascular diseases (CVD), including atherosclerosis. FIP has been found to occur not only in individuals with general obesity but also in those without established pancreatic disease. It has been strongly associated with diabetes and severe widespread atherosclerosis (102, 103). FIP has been linked to a higher prevalence of carotid artery plaques and increased vascular rigidity in individuals who are not overweight. Nonetheless, no significant associations were found in obese individuals (104).

Studies have also shown that FIP is linked with augmented epicardial adipose tissue and aortic intima-media thickness, which are markers of clinical atherosclerosis. FIP was strongly associated with a 3-fold increased risk of developing aortic intima-media thickness and a 16-fold increased risk of developing epicardial adipose tissue (105). Furthermore, FIP has been correlated with the severity of coronary artery stenosis in individuals with T2DM, suggesting that it may be a predictor of coronary artery stenosis in this population. A higher incidence of complex coronary artery lesions was found in patients with acute coronary syndrome (ACS) and FIP. A significant and independent positive correlation was observed between FIP and the Syntax (SX) score, a well-established angiographic scoring system considering lesion characteristics and coronary anatomy (106).

The pathophysiological mechanisms underlying the association between FIP and coronary atherosclerosis involve the secretion of pro-inflammatory factors by adipose tissue in the pancreas, consequently resulting in endothelial dysfunction and metabolic abnormalities. The severity of fat accumulation in the pancreas has also been correlated with the severity of coronary arteriosclerosis. These findings suggest that FIP may be a potential contributor to CVD, particularly in individuals who are not overweight (107).

### 7.4. FIP and HTN

HTN is a significant global health issue associated with the risk of heart, brain, kidney, and other organ disorders, and it is the leading cause of death worldwide.

A study involving 55 non-diabetic human pancreas donors revealed that donors with a history of HTN had higher pancreatic fat and islet fat content, regardless of gender. These findings suggest that a previous history of HTN may independently contribute to increased pancreatic fat content and islet fat content (108). HTN is known to cause small vessel constriction and a reduction in microvascular density, leading to decreased tissue perfusion (109). Hypoxia and hypoxia-inducible factors have been linked to alterations in lipid metabolism and intracellular lipid accumulation in cultured cells and organs such as the liver and pancreatic islets (110, 111). Consequently, HTN may contribute to persistent tissue hypoxia, leading to lipid accumulation and pancreatic islet dysfunction.

On the other hand, a meta-analysis study reported a 67% increased risk of HTN associated with FIP (13). However, a prospective study showed a non-statistically significant trend toward an association between HTN and FIP (5). In a cross-sectional retrospective study involving 65 children with NAFLD, FIP was 1.28 times more likely to cause HTN (OR 1.28, 95% CI: 1.01–1.62) when examining potential risk factors for HTN in children with NAFLD (112).

### 7.5. FIP and T2DM

T2DM is projected to become the sixth leading cause of mortality by 2030, with obesity and physical inactivity being significant contributors to its development, and it is crucial to understand their impact on the pancreas and associated morbidity.

The build-up of ectopic fat in non-adipose tissues, such as the pancreas, due to metabolic overload of adipose tissue may result in lipotoxicity, insulin resistance, and inflammation, leading to glucose metabolic disturbances and T2DM. The build-up of fat in pancreatic endocrine cells plays a crucial role in the pathogenesis of T2DM (113). FIP has been implicated in the pathogenesis of T2DM, even independent of NAFLD, with evidence suggesting that it may be associated with beta-cell failure independent of insulin resistance (114, 115). However, other studies have reported that NAFLD patients with FIP have increased insulin resistance, impaired glucose parameters, and higher rates of prediabetes and diabetes than those with NAFLD alone (116). In an animal study, pancreatic fat build-up was observed before the onset of hyperglycemia in obese Zucker diabetic fatty mice (117). A cross-sectional study also reported a significantly higher prevalence of T2DM in FIP patients compared to non-FIP patients (12% vs. 5%) (7).

In some studies, pancreatic fat content, measured by various methods such as MRS, MRI, and CT, is higher in individuals with T2DM than in non-diabetic subjects. However, some studies using CT or post-mortem examinations have reported no difference in pancreatic fat content, and the discrepancies in findings are due to differences in measurement accuracy (116).

A meta-analysis study revealed that individuals with FIP had a 108% higher risk of developing T2DM. Another meta-analysis of six studies showed that individuals with FIP had a significantly increased relative risk of developing T2DM (RR 2.08; 95% CI: 1.44–3.00; *P* = 0.001). Some studies have also reported a significant association between FIP, insulin resistance, and beta-cell dysfunction (20).

Recently, a 10-year prospective cohort study involving 631 participants found that those with FIP had a significantly higher incidence of T2DM than those without FIP (33.3% vs. 10.4%; *P* < 0.001). FIP was independently associated with an increased hazard ratio of 1.81 for incident T2DM. Furthermore, the risk of developing incident T2DM was significantly increased by 7% for every 1% increase in pancreatic fat, with a hazard ratio of 1.07 (35).

However, recent studies have shown that T2DM may be reversible with bariatric surgery and dietary energy intake restriction, which can improve beta-cell activity and reduce pancreatic fat content.

The role of FIP, particularly the first-phase insulin response, may be crucial in glucose metabolism and beta-cell failure, and different susceptibility thresholds to pancreatic fat build-up may be determinants of beta-cell dysfunction (118).

### 7.6. FIP and PF

Pancreaticoduodenectomy (PDE) is currently the preferred treatment for pancreatic cancer. However, one of the most significant complications of PDE is PF. Early detection and management of potential risk factors for PF are crucial. FIP has been identified as a significant risk factor for PF (119). Studies have shown that patients with PF have significantly greater intra-lobular, inter-lobular, and total pancreatic fat than non-PF patients.

Moreover, the risk of PF becomes considerable when the percentage of fatty infiltration exceeds 10% (26, 119). In a retrospective study, 40 patients with PF and 40 without PF were selected and matched for age, sex, pancreatic pathology, surgeon, and type of surgery. Compared to non-PF patients, those with PF demonstrated a considerable increase in intra-lobular, inter-lobular, and total pancreatic fat content (*P* < 0.001). An inverse correlation was observed between PF and fibrosis, blood vessel density (*P* < 0.001), and a small PD. A soft pancreatic texture is also deemed a risk factor for PF formation, potentially owing to augmented fat build-up, which can impede anastomosis and heighten the likelihood of perioperative pancreatitis (120, 121).

A meta-analysis of 11 studies involving 2484 individuals found that FIP was significantly associated with the occurrence of the PF (OR = 3.75; 95% CI: 1.64, 8.58; *P* = 0.002; *I*^2^ = 78). Overall, the evidence suggests that FIP is a critical risk factor for PF and should be carefully managed during PDE to reduce the incidence of PF (119). The exact mechanism by which FIP promotes PF development is unknown. Nonetheless, researchers propose that a soft pancreas is more susceptible to ischemia and damage during restoration, and the exocrine function can worsen tissue damage (122).

### 7.7. FIP and pancreatic transplantation

Pancreatic transplantation has become increasingly successful, and as a result, the number of individuals awaiting pancreatic transplantation has risen. FIP is an essential criterion in determining the viability of donor pancreata for transplantation. Although many pancreas from overweight donors can be transplanted successfully, certain transplant surgeons avoid using the pancreas with significant FIP since the surgery is technically more challenging. However, a more objective measurement could help avoid discarding suitable organs for transplant. In the case of pancreatic transplantation, the presence of pancreatic lobules with associated ducts and blood arteries loosely separated from the primary pancreas recommends that all cut-away fatty pancreatic tissue be ligated to prevent pancreatic leaks and bleeding after transplantation. Careful hemostasis after fat removal minimizes the chance of hematoma leading to infection, especially when anticoagulation is indicated, even minor anticoagulation. To prevent fat necrosis, octreotide should be administered prior to revascularization and continued until the pancreatitis is resolved, rather than late after surgery. Flushing and cooling should be used to maximize graft preservation and avoid autolysis (78).

### 7.8. FIP and PDAC

PDAC is a highly lethal cancer worldwide (123, 124). The late presentation and lack of early-stage biomarkers make early identification of PDAC challenging. Additionally, the retroperitoneal location of the pancreas facilitates the spread of PDAC to nearby organs and blood vessels, and non-specific features further complicate early detection (125).

PDAC typically arises from precursor lesions such as acinar-ductal metaplasia (ADM), pancreatic intraepithelial neoplasia (PanIN), intraductal papillary mucinous neoplasia (IPMN), mucinous cystic neoplasia, and atypical flat lesions (126, 127). The most common genetic alteration in PDAC is the KRAS mutation, which is detected in more than 90% of cases and is an early event in low-grade PanIN 1A lesions (126). Obesity is a significant risk factor for PDAC (128, 129), and increased pancreatic adiposity contributes to PDAC development from FIP (130). Animal studies have shown that obesity and hyperlipidemia enhance FIP, leading to the progression of N-nitrosobis(2-oxopropyl)amine (BOP)-induced PDAC and the upregulation of adipocytokines and cell proliferation-related genes in the pancreas (131). Thus, local adipocytokine production from adipose tissues in an adipose tissue-rich milieu is linked to the development of PDAC.

PanIN, which affects small pancreatic ducts, may contribute to localized pancreatitis and promote the development of neoplasms. FIP is linked to the development of PanIN, and it is the most significant risk factor for precancerous pancreatic lesions, according to a retrospective study with an odds ratio of 17.86 [4.94–88.12], independent of age and diabetes status (132).

A case–control study of patients who had surgical intervention for PDAC found that increased pancreatic fat enhanced PDAC propagation and mortality. Fat-induced aberrant local cytokine production and toxic fatty acids have been shown to play a role in tumor proliferation, invasion, and angiogenesis (133). Additionally, persistent overproduction of reactive oxygen species can cause and accelerate mutagenesis alterations that contribute to the development and progression of PDAC (134). These mechanisms and the generation of growth factors by adipocytes are likely to hasten the spread of cancer cells to the lymphatics and local lymph nodes. FIP affects the tumor microenvironment, accelerates tumor progression, and leads to the early death of individuals with PDAC (135).

### 7.9. The new clinical consequences of FIP

Polycystic ovary syndrome (PCOS) is the most prevalent endocrine disorder impacting women during their reproductive years (136). Osman et al. found that FIP was identified in 38.0% of patients with PCOS, significantly higher than the 12.0% of FIP cases detected in healthy controls (1). On the other hand, a recent study on adolescents did not find a relationship between FIP and PCOS (137). Notably, Osman et al. found that age was independently associated with the development of FIP in PCOS patients, suggesting that the metabolic interactions between PCOS and FIP may take longer to become evident in younger patients. The complex connection between FIP and PCOS reveals the intersection of metabolic and endocrine issues. Ongoing research could transform our grasp of these conditions and lead to innovative treatments.

## 8. Treatments of FIP

Until recently, there was no conventional treatment for FIP. However, lifestyle modifications such as weight loss, a healthy diet, and regular exercise have shown promise in reversing FIP by lowering calorie intake. Studies conducted on animal and human models have demonstrated the efficacy of weight loss through bariatric surgery in reversing FIP and its clinical consequences, including T2DM.

Pharmacological therapy for FIP has also been investigated in animal studies, with oral hypoglycemic medications such as metformin, dipeptidyl peptidase-4 inhibitors (DPP-4 inhibitors), and thiazolidinediones showing promise in managing FIP in patients with T2DM. Metformin, a first-line anti-diabetic medication, has been found to improve lipid metabolism and directly affect insulin secretion in pancreatic islets by reducing oxidative stress. It can also reduce visceral fatty tissue by improving insulin resistance and suppressing compensatory beta-cell hyperplasia resulting from a high-fat diet (138).

In animal studies, Troglitazone administration significantly increased pancreatic weight and protein content, effectively preventing or reversing inflammatory cell infiltration, pancreatic fatty replacement, and fibrosis in T2DM mouse models. Pancreatic lipase inhibition through tetrahydrolipstatin decreased the severity of acute inflammation of the pancreas, fat apoptosis, pancreatic failure, and mortality in obese mice with FIP. Moreover, the combined use of Sitagliptin and Telmisartan, two anti-diabetic medications, showed efficacy in managing FIP and preventing the progression from lipotoxicity to severe pancreatic disease in mice. Berberine and cinnamic acid, components of a novel Japanese drug known as the “Jiaotai Pill,” were found to inhibit lipid accumulation in pancreatic beta-cell cultures by lowering lipogenesis and boosting lipid oxidation (139).

In obese rats, Sandostatin (a somatostatin analog) downregulated the expression of an adipose differentiation-associated protein in the pancreas, reducing pancreatic fatty deposition, lipid dysregulation, and insulin resistance and ameliorating pancreatic damage (140).

Although these findings hold promise for FIP treatment, further long-standing interventional research is necessary to establish the mortality benefit, improvement in glycemic management, and incidence of pancreatitis and pancreatic cancer for prospective therapeutic options.

## 9. Conclusion

FIP is a common yet frequently overlooked disorder that requires increased attention and investigation to comprehend its clinical significance. Early detection and understanding of FIP may aid in developing and strengthening management strategies, leading to improved patient clinical outcomes. Recent studies suggest a higher prevalence of FIP in patients with PCOS, with age being independently associated with its development. This highlights the intriguing crossroads of metabolic and endocrine dysfunction. Early screening for FIP may facilitate early detection of PDAC, the lethal clinical consequence of FIP. However, there are currently no suitable biomarkers for FIP detection, and diagnosis relies on histopathology and imaging. A simplified scoring system developed by Khoury et al. has demonstrated high accuracy and specificity in identifying the presence of FIP. Management guidelines for FIP are yet to be established and require further research.

## Author contributions

MAM, ME, YJ, and PZ contributed to the conception of the review. MAM conceived the study subject and made substantive revisions to the important content of the manuscript, and he was the major contributor to the writing of the manuscript. AM, MA, MAlm, and MAM provided suggestions and technical support, revised important manuscript sections, and assisted in the literature search. JW, MAln, PZ, and SH critically reviewed the manuscript. All authors contributed to the article and approved the submitted version.
